# Determining the Topic Evolution and Sentiment Polarity for Albinism in a Chinese Online Health Community: Machine Learning and Social Network Analysis

**DOI:** 10.2196/17813

**Published:** 2020-05-29

**Authors:** Qiqing Bi, Lining Shen, Richard Evans, Zhiguo Zhang, Shimin Wang, Wei Dai, Cui Liu

**Affiliations:** 1 School of Medicine and Health Management Tongji Medical College Huazhong University of Science & Technology Wuhan China; 2 Hubei Provincial Research Center for Health Technology Assessment Wuhan China; 3 Institute of Smart Health Huazhong University of Science & Technology Wuhan China; 4 College of Engineering, Design and Physical Sciences Brunel University London London United Kingdom

**Keywords:** albinism, rare diseases, topic mining, social network analysis, sentiment polarity, online health community, machine learning

## Abstract

**Background:**

There are more than 6000 rare diseases in existence today, with the number of patients with these conditions rapidly increasing. Most research to date has focused on the diagnosis, treatment, and development of orphan drugs, while few studies have examined the topics and emotions expressed by patients living with rare diseases on social media platforms, especially in online health communities (OHCs).

**Objective:**

This study aimed to determine the topic categorizations and sentiment polarity for albinism in a Chinese OHC, Baidu Tieba, using multiple methods. The OHC was deeply mined using topic mining, social network analysis, and sentiment polarity analysis. Through these methods, we determined the current situation of community construction, identifying the ongoing needs and problems experienced by people with albinism in their daily lives.

**Methods:**

We used the albinism community on the Baidu Tieba platform as the data source in this study. Term frequency–inverse document frequency, latent dirichlet allocation models, and naive Bayes were employed to mine the various topic categories. Social network analysis, which was completed using the Gephi tool, was employed to analyze the evolution of the albinism community. Sentiment polarity analysis was performed using a long short-term memory algorithm.

**Results:**

We identified 8 main topics discussed in the community: daily sharing, family, interpersonal communication, social life and security, medical care, occupation and education, beauty, and self-care. Among these topics, daily sharing represented the largest proportion of the discussions. From 2012 to 2019, the average degree and clustering coefficient of the albinism community continued to decline, while the network center transferred from core communities to core users. A total of 68.43% of the corpus was emotional, with 35.88% being positive and 32.55% negative. There were statistically significant differences in the distribution of sentiment polarity between topics (*P*<.001). Negative emotions were twice as high as positive emotions in the social life and security topic.

**Conclusions:**

The study reveals insights into the emotions expressed by people with albinism in the Chinese OHC, Baidu Tieba, providing health care practitioners with greater appreciation of the current emotional support needed by patients and the patient experience. Current OHCs do not exert enough influence due to limited effective organization and development. Health care sectors should take greater advantage of OHCs to support vulnerable patients with rare diseases to meet their evidence-based needs.

## Introduction

### Background

Rare diseases are considered conditions that affect a limited amount of people, typically less than 1 in 2000 individuals. Albinism is a type of rare disease related to a variable hypopigmentation phenotype, where patients experience partial or complete absence of pigment in their skin, eyes, and hair [1]. Despite advances in genomic technology and medicines, many individuals affected with rare diseases remain undiagnosed, and some never receive a definitive diagnosis [2]. A diagnosis with a rare disease is extremely likely to cause economic, psychosocial, and physical burden on the patient and family members [3]. Research demonstrates that parents of children with rare genetic disorders present feelings of social isolation, anxiety, fear, anger, and uncertainty [4] and experience high levels of physical and emotional strain [5].

### Related Research

Over the last decade, rare disease research has received considerable attention in health care studies, with exploration typically focusing on 1 of 3 main areas: etiology, diagnosis, and treatment [6]. In recent years, rare disease research has also straddled other disciplines, including policy improvement, sociology, psychology, and ethics. For example, Abbas et al [7] reported that the European Union and United States have adopted policies and regulations aimed at improving orphan drug availability over the past 20 years, but that only 16 countries had an orphan drug or rare disease plan in place. Rodwell and Ayme [8] reviewed the political frameworks of European countries to demonstrate how legislation has created a dynamic that is progressively improving health care for patients with rare diseases. Dharssi et al [9] found that patient communities are being used to promote and drive the establishment and adoption of legislation and programs to improve rare disease care. Gomes [10] discussed the construction of social identity, mutual recognition, and the specific demands for recognition of people with rare conditions from 3 sociological perspectives.

### Online Health Communities

Online health communities (OHCs) have become a popular means for individuals to obtain support and connect with others online when experiencing illness, especially patients with similar diagnoses [11]. An increasing amount of literature related to OHCs documents widespread concerns from scholars worldwide. Some researchers have focused mostly on social networks and user behaviors. For example, Huh et al [12] conducted open coding analysis using interview data and cluster analysis to determine that 4 types of persona exist in OHCs: caretakers, opportunists, scientists, and adventurers. Lu el al [13] investigated health care social media use from different stakeholder perspectives using content analysis. Others have concentrated on knowledge sharing and value creation. For example, Yan et al [14] proposed a benefit versus cost knowledge sharing model for OHCs. Guo et al [15] conducted an empirical investigation into the relationship between professional capital and exchange returns in OHCs. In addition, health interventions have been reported based on OHCs. Naslund et al [16] established that people with serious mental health illnesses reported benefits from interacting with peers online, experiencing greater social connectedness. Most existing OHC research has examined chronic diseases, such as cancer, diabetes, AIDS, and severe mental disorders, using large patient populations and relating more to social concerns [17-20]. Furthermore, social media tools have been studied, such as Wechat Official Accounts [21] and SentiHealth-Cancer [22]. However, there are few studies that have focused on OHCs for rare diseases. Davies et al [23] found that online surveys for stakeholder groups may provide new insights into rare conditions and their management relatively quickly, with the possibility of rapid translation into health care intervention management and policy development. Although the number of patients with rare diseases is limited, some scholars have pointed out that patients with such conditions require increased social support networks [24].

### Objectives

The main type of albinism is oculocutaneous albinism, which is a group of conditions that affect the coloring (pigmentation) of the skin, hair, and eyes. Long-term exposure to the sun can greatly increase the risk of skin damage and cancer [25]. Melanin deficiency causes a series of abnormalities in the eyes, such as severe low vision, photophobia, and nystagmus. Due to its special phenotype, the psychological development of patients with albinism is affected [26]. The worldwide prevalence of oculocutaneous albinism is estimated to be 1 in 17,000 [27]. In the Chinese Han ethnic group population of the Shandong province in China, the prevalence is approximately 1 in 18,000, or roughly 3.80% of the population [28]. In addition to the general characteristics of more typical rare diseases, albinism has a certain uniqueness and patient base. Current academic research into albinism has focused on etiology [29], pathology [30,31], diagnosis [32-35], sociology [36,37], and albinism in animals [38,39].

To our knowledge, no studies exist on albinism-based OHCs, aimed at deeply detecting the prevailing topics, their change over time, and sentiment polarity (ie, sentimental expressions of albinism patients and the distribution of different sentiments). This study aimed to guide the academic community to focus more on rare diseases in albinism OHCs. Specifically, this study aimed to answer 3 research questions. What is the topic evolution for albinism in OHCs? What are the characteristics of albinism social networks in OHCs? What is the sentiment polarity of albinism in OHCs?

## Methods

### Sample and Data Collection

**Figure 1:**
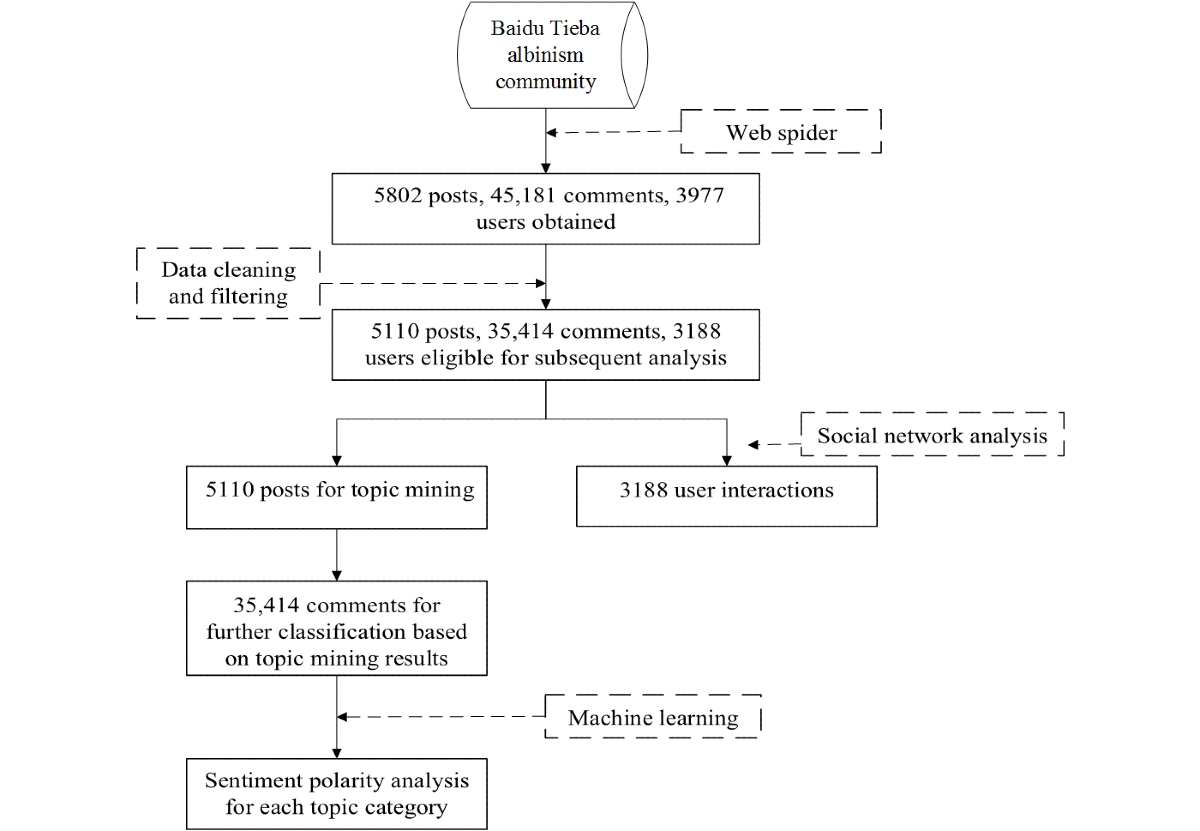
Flowchart for identifying data from the online albinism Baidu community for subsequent analysis.

Few OHCs for albinism exist in China, with most related to social media, such as Tencent QQ, WeChat, and Baidu Tieba [40]. Baidu Tieba is the largest Chinese communication platform for discussion and the posting of questions [41], with data being readily available and considered high quality. This platform contains millions of online communities targeted at specific topics. The Baidu albinism community has over 300,000 registered users. Accordingly, we designed a web spider using Python 3.7 [42] Scrapy [43] to crawl the records dated from January 30, 2007 to March 14, 2019, including a total of 5802 posts, 45,181 comments, and 3977 active users. The dataset contains content of posts and the complete text of comments, as shown in Textbox 1. Given that some data collected before 2012 were severely lost and fragmented, the dataset from 2012 to 2019 was eventually selected for subsequent analysis. In addition, the following user-posted content was also removed: non-text content (eg, video, music, picture) or content with missing author and time fields. The final dataset included 5110 posts, 35,414 comments, and 3188 active users. The process for identifying data for subsequent analysis is shown in Figure 1. Moreover, we categorized users who had not used the albinism community for more than 1 year as “lost users,” and users who had used the community more regularly as “new users.”

**Data fields extracted from the online albinism community.**

**Albinism_Post**

- Post_id (post id)
- Post_title (post title)
- Author_id (author’s id)
- Content (post content)
- Time (post time)
- Reply_num (number of replies)
- URL (URL of the post)

**Albinism_Comment**

- Comment_id (comment id)
- Post_id (post to which the comment belongs)
- Author_id (author’s id)
- Content (comment content)
- Time (comment time)
- Floor (the floor in its post, which represents a comment from a user, and the floor number is order of user comments)

### Data Analysis Methods

#### Topic Mining

To ensure the amount and accuracy of topic mining, this study used the title and comments as the topic mining corpus. After data cleansing, the dataset for topic mining contained 10,220 corpora. First, Jieba 0.39 [44] in Python 3.7, the Chinese word segmentation tool, was employed for word segmentation. Owing to the particularity of albinism in the medical field, we used the International Statistical Classification of Diseases and Related Health Problems, 10th Revision and Chinese Medical Subject Headings to expand the lexical dictionary for intervention. In addition, based on the stop word list of the Harbin Institute of Technology in China, our stop word list was continuously updated through the results throughout the experiment.

Then, we combined term frequency–inverse document frequency and latent dirichlet allocation (LDA) [45] for topic mining; the number of topics was identified based on the perplexity [46]. Here, LDA, the most common method for topic modelling, is a generalization of probabilistic latent semantic indexing [47]. Perplexity is a common criterion for evaluating the effectiveness of language models [48]. Due to each topic in the LDA results containing multiple types of topic information, two research assistants (RAs) with medical backgrounds were hired to independently annotate each LDA category with 1-3 labels. Then, the RAs evaluated the results independently to reach consensus, with discussions for any discrepancies or disagreements joined by the first author of this study. Subsequently, the assigned labels were combined, deduplicated, and reclassified to form the final classification label. Moreover, a naive Bayes (NB) model was used, which performs well with small-scale data and can handle multiple classification tasks commonly used for text classification [49]. Therefore, on the basis of the new classification label, a NB classifier was created to classify all posts, with a precision rate of 0.889, recall rate of 0.915, and F1 score of 0.902. Finally, each comment was merged into the topic of the corresponding post; the topic classification for the full corpus was implemented since the comment text was short and the topic information was limited.

#### Social Network Analysis

A social network is the integration of social relationships. With the increase in popularity of social media sites, scholars and practitioners have aimed to understand the behaviors of people using such platforms [50,51]. Gephi, a social network visualization software, is used in various disciplines. One of its key features is the ability to display the spatialization process [52]. Gephi 0.9.2 [53] was employed in this study to analyze the topology of the interaction between 3188 users, based on the community mining algorithm built in the software [54], which can detect the potential community of users. As the results of the analysis for all user data were ambiguous, we identified a 2-year interval to explore the dynamic evolution of the community structure to better reflect the users’ activity. To better reflect the social network characteristics of the albinism bar, we compared it to the random networks with the same number of nodes based on several basic indicators, including average degree, network diameter, number of communities, clustering coefficient, and average path length. The average degree represents the average distance between nodes. The clustering coefficient is a coefficient indicating the degree of node aggregation in a graph. The average path length is the average shortest distance between all pairs of nodes in the network.

#### Sentiment Polarity Analysis

Sentiment polarity analysis, commonly used in academia, mainly includes a sentiment dictionary and machine learning. And the frontier branch of machine learning is deep learning [55,56]. At present, the enhanced version of machine learning algorithms is widely used in sentiment analysis [57,58]. Therefore, we selected 4 representative training classifiers of machine learning algorithms, including NB, support vector machine, convolutional neural network, and long short-term memory. Sentiment polarity was divided into 3 polarities: positive, neutral, and negative. We first randomly chose more than 4000 corpora and then marked them with one of these 3 sentiment polarities using Colabeler (Hangzhou Kuaiyi Technology Co Ltd, Hangzhou, Zhejiang, China), a labeling program. Then, we selected 1000 records marked with one sentiment polarity from 4000 corpora for the sentiment classification model training. The corpus that stated objective facts was marked as neutral. The others that contained obvious sentiment words and emotions were marked as positive or negative. In this process, we referred to the Hownet sentiment lexicon [59] from the China National Knowledge Infrastructure and the Chinese sentiment lexicon and sentiment analyzer from the National Taiwan University School of Dentistry [60]. As shown in Table 1, the long short-term memory classifier performed best in the testing of sentiment polarity for the remaining corpora, in comparison with the 3 alternative machine learning algorithms. Finally, the differences in sentiment distribution between topics was verified using a Chi-square test executed in SPSS 20.0 (IBM Corp, Armonk, NY).

**Table 1:** Performance of the models for sentiment polarity classification.

	Precision	Recall	F1 score
NB^a^	0.798	0.835	0.816
SVM^b^	0.853	0.822	0.837
CNN^c^	0.801	0.823	0.812
LSTM^d^	0.916	0.916	0.916

^a^NB: naive Bayes.

^b^SVM: support vector machine.

^c^CNN: convolutional neural network.

^d^LSTM: long short-term memory.

## Results

### Basic Statistical Information

**Figure 2:**
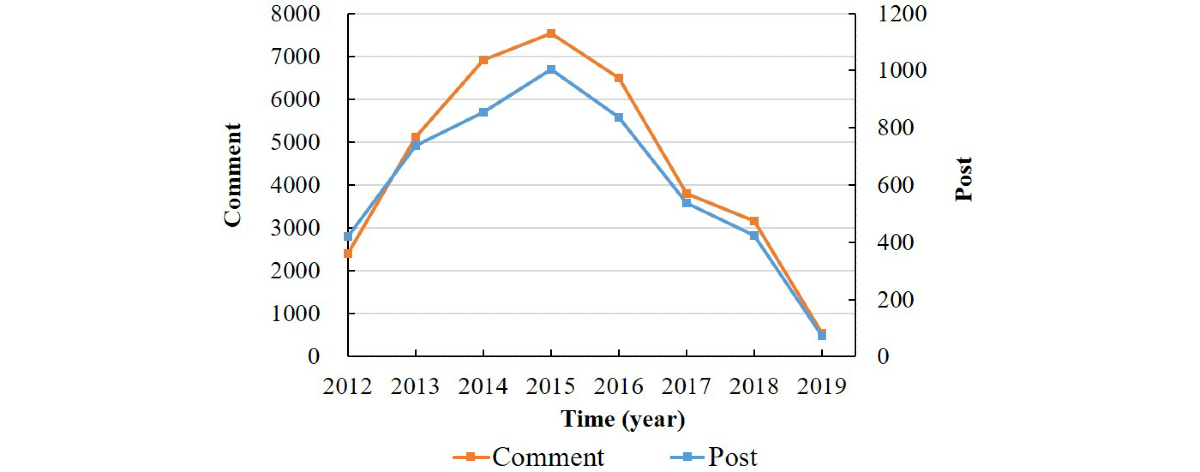
Posts and comments about albinism in the online community in 2012-2019.

**Figure 3:**
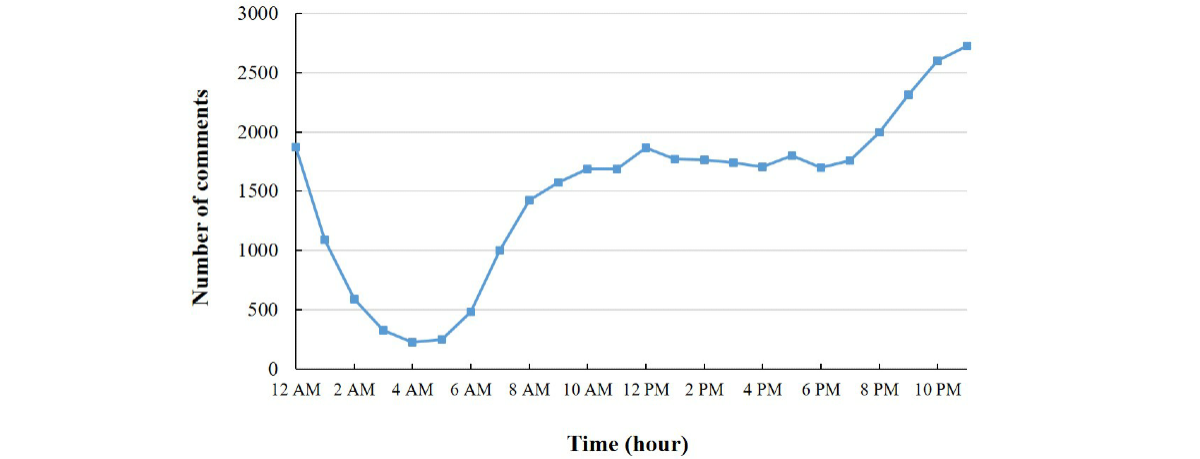
Distribution of the comments in the online albinism community by hour of the day.

From 2012 to 2019, the number of posts and comments showed the same trend: they increased during the early years of the study, reached a peak in 2015, and subsequently declined (Figure 2). The findings revealed that the users preferred to use the albinism community after 6:00 pm, with all other times similar in frequency of use; there were only two small peaks at lunch and dinner times, as shown in Figure 3.

**Figure 4:**
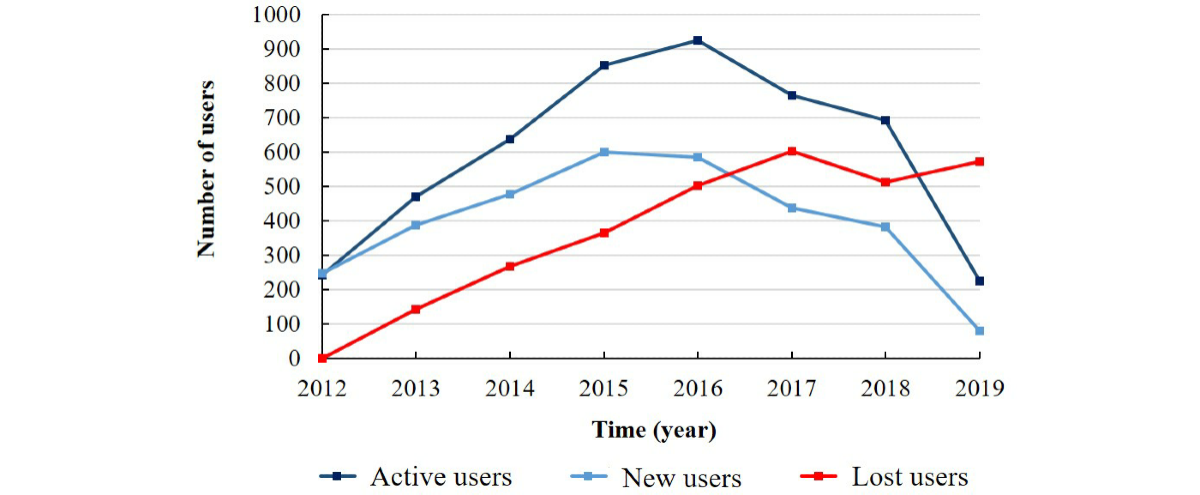
Number of users in the online albinism community per year.

**Figure 5:**
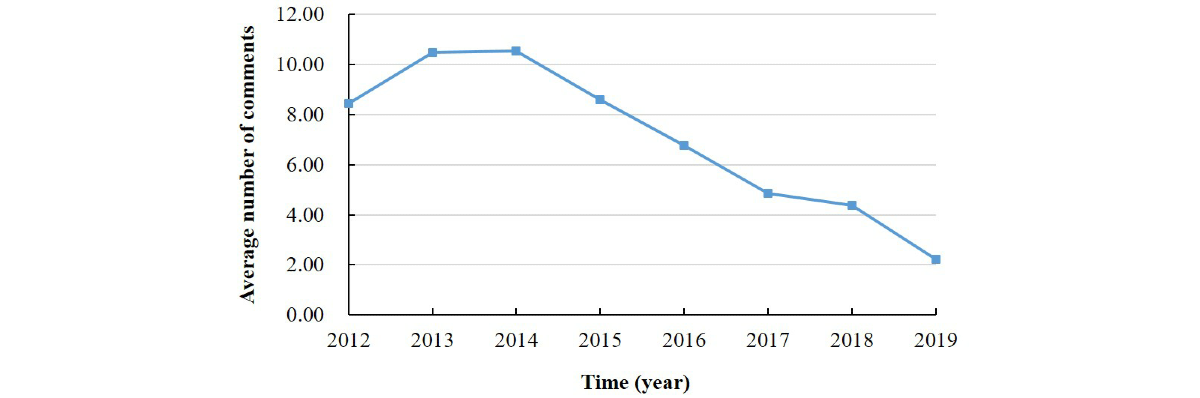
Average number of comments posted in the albinism community each year.

Figure 4 shows that the number of active users increased during the early years of the study period but peaked in 2016 and then declined. Furthermore, the number of “lost users” increased each year, indicating that the speed of user abandonment increased, whereas the number of “new users” increased at the beginning and then decreased at a faster rate than it increased. The superposition of the two curves shows a significant decline in the number of active community members. The trend remained obvious even after omitting the 2019 data. Figure 5 presents the average number of posts submitted by users each year, showing a decreasing trend year by year.

### Topic Evolution

**Figure 6:**
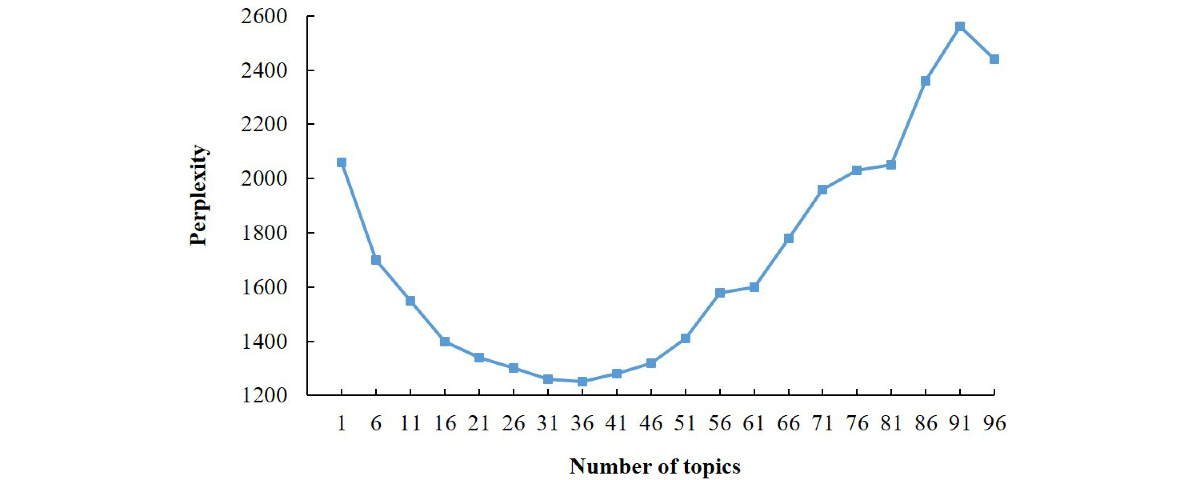
Latent dirichlet allocation model topic number in a perplexity diagram.

As shown in Figure 6, the lowest perplexity was 36, which determined the value of the parameter num_topics of the LDA document topic generation model. For the details of these 36 categories, see Multimedia Appendix 1. Moreover, after merging and sorting, the final classification labels were formed, with a total of 8 categories, shown in Table 2.

**Table 2:** The resulting 8 categories for the posts about albinism in the online community.

Number	Category name	Description	Examples
1	Daily sharing	Sharing of daily life experiences (not included in topics 2-8)	The weather is really good today!
			It's unlucky to lose money.
2	Family	Sharing of daily life experiences from the perspective of family members of people with albinism	I have an angel baby.
			My child is diagnosed with albinism, so desperate.
3	Interpersonal communication	Social contact requests	Let's make friends!
			Are there friends from Beijing? This is my QQ number.
4	Social life & security	Discussion of social impact or social commonality	How do I apply for a disability certificate?
			Where can I get free vision glasses?
5	Medical care	Medical issues, such as treatment, examination, and protection	What medical examination is needed?
			What about nystagmus?
6	Occupation & education	Issues related to occupation or education	How about the income of the massage industry?
			Does albinism not affect school?
7	Beauty	Issues related to hair care, dyeing of hair, or makeup	Can people with albinism dye their hair?
			The younger sister's makeup is really beautiful.
8	Self-care	Other issues related to daily life (not included in topics 3-7)	How to repair the computer?
			How to register a game account?

After all the comments were classified as topics according to the results of the topic category of the posts, the daily sharing category accounted for the largest proportion (17,010/35,414, 48.03%) of the total comments, indicating that users were open to expressing their feelings and daily life through social media. Medical care was the second most common subject discussed by users, accounting for 12.04% (4264/35,414) of the total comments posted. With regards to this category, genetic testing, prenatal testing, vision protection, skin protection, and treatment were the major topics discussed. An indepth analysis of the corpus found that users were confused about methods of protection and how to obtain them. Interpersonal communication was the third most discussed topic among users, accounting for 11.20% (3966/35,414) of the comments posted. This reflects the social attributes of Baidu Tieba, with users searching for suitable companions based on region, age, hobby, and disease severity. There were also numerous exchanges in the occupation & education category, representing 10.53% (3729/35,414) of the total comments; these two aspects were observed to be a severe annoyance for people with albinism. Visual impairment and fragile skin interfere with occupation and education. The family and beauty categories accounted for 6.17% (2185/35,414) and 5.00% (1771/35,414), respectively, of the posted comments. The family category reflected the emotional expression among family members. As the issues for family members are also involved in the medical care and social life & security aspects for people with albinism, the proportion here is slightly lower. Beauty reflected the patient’s pursuit of appearance and positive attitude towards life, which can alleviate some practical issues. The categories with the lowest number of comments were social life & security (1558/35,414, 4.40%) and self-care (931/35,414, 2.63%). The social life & security category included public welfare activities, public events, policies, and regulations, representing the maintenance of patients’ rights and interests.

**Figure 7:**
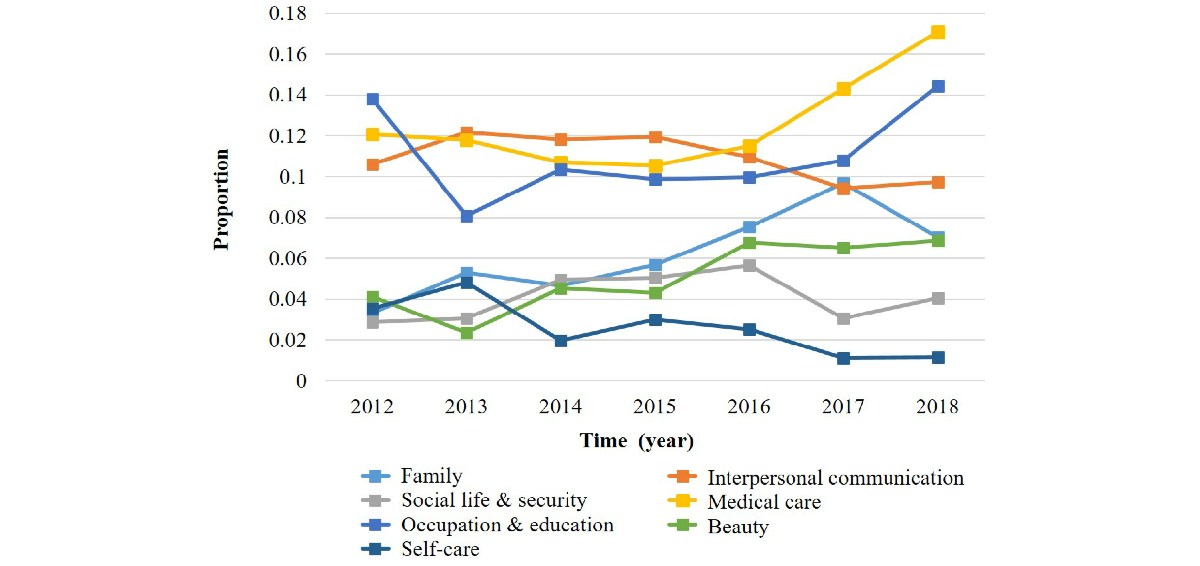
Topic evolution by year, with each category reported as a proportion of the total comments per year.

The absolute number of each topic corpus was affected by the overall trend. Figure 7 shows the change in the proportion of 7 topic categories from 2012 to 2018; the daily sharing category was excluded because its proportion far exceeded those of the other categories. It can be intuitively seen that the number of posts within the medical care, occupation & education, and beauty categories dynamically increased during the study period. Among the categories, the increase in the number of posts in the medical care category is the most obvious. These 3 categories represent a certain degree of disease experience sharing, indicating that the online albinism community provided an effective platform for patients to solve problems to some extent. The number of posts in the family category also experienced an upward trend but declined in 2018. The number of posts in the other 3 categories fluctuated or declined to varying degrees during the study period.

### Social Network Structure

As shown in Table 3, we observed that the average degree and clustering coefficient continued to decrease, while the network diameter, number of communities, and average path length increased. However, these results are better than that of random networks with the same size from the perspective of user interaction. This shows that there is a small world effect between users, which can form effective communication, but this effect is gradually decreasing.

**Table 3:** Basic statistics for the social network analysis, compared with those of a random network.

Year	Number of users	Average degree		Network diameter		Number of communities		Clustering coefficient		Average path length	
		Study network	Random network	Study network	Random network	Study network	Random network	Study network	Random network	Study network	Random network
2013	629	5.67	16.08	7	10	6	8	0.210	0.026	3.20	2.58
2014	951	7.00	23.70	8	10	9	10	0.176	0.025	3.21	2.45
2015	1268	6.78	31.73	9	10	13	11	0.136	0.025	3.36	2.36
2016	1472	5.99	36.98	8	9	13	10	0.113	0.025	3.51	2.31
2017	1415	4.81	35.46	10	10	13	10	0.097	0.025	3.81	2.32
2018	1212	3.98	30.37	11	9	16	10	0.077	0.025	4.35	2.37
2019	796	3.29	19.79	14	11	23	10	0.072	0.025	4.59	2.49

**Figure 8:**
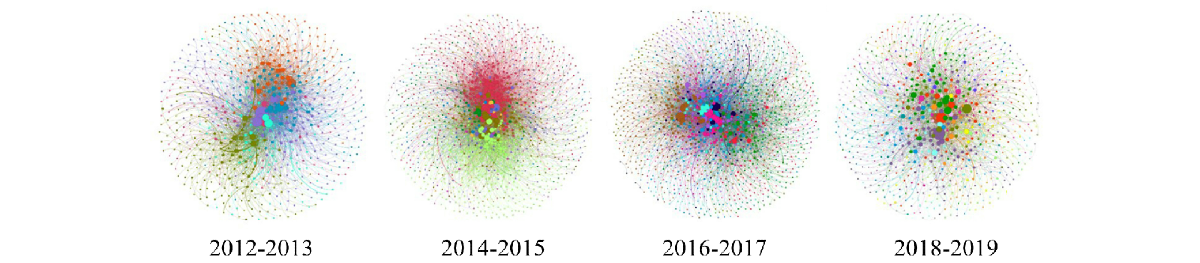
Changes in the community structure over time.

Figure 8 presents the evolution of the community structure from 2012 to 2019, which reflects the distribution characteristics of core edge. The node represents the users, and the node size is proportional to the degree. Different communities are distinguished by color. The edge represents the comment relationship between users. The structural changes occurred from the core community to the core user as the principal part in evidence. From 2012 to 2016, the number of communities increased in the central region. Meanwhile, the scale expanded, and the structure matured. From 2016 to 2019, the community replaced by core users has become blurred in the central region, while the number of core users has increased significantly.

### Distribution of Sentiment Polarity

Daily sharing was the most active category (12,581/17,010, 73.96%) for expressing emotions, with positive emotions being observed the most often (7170/17,010, 42.15%), as shown in Table 4. When users encounter events that affect their emotions in their daily lives, they tend to vent through social media. The online albinism community is seen to provide a platform for confiding with other people with albinism and their families. In addition, the medical care category had the highest proportion (1671/4264, 39.19%) of negative emotions. Most people with albinism have skin and vision dysfunction, which causes a number of practical issues that affect quality of life. The negative emotions expressed in the medical care category arose from issues mainly related to anxiety and worry, such as “Does this disease only affect white-skinned people?” and “How do I deal with blurred vision?” With regards to the family category, there were many similar statements such as “I cry at home every day” or “I don’t know what to do” that conveyed feelings of sadness, confusion, and helplessness. Moreover, the social life & security category had a high proportion of negative emotions (588/1558, 37.74%), twice that of the number of positive emotions. This category is concerned mostly with public benefits such as the distribution of visual aids, health education, and offline activities. However, many posts referred to the handling and grading of disability certificates, social discrimination issues, and medical insurance, all of which are likely to increase negative emotions. In addition, the statistical test results showed a statistically significant difference in the distribution of sentiment polarity between topic categories (χ^2^_14_=1083.368, *P*<.001).

**Table 4:** Results of the sentiment polarity analysis results for the 8 topic categories.

Topic category	Positive, n (%)	Neutral, n (%)	Negative, n (%)
Daily sharing	7170 (42.15)	4429 (26.04)	5411 (31.81)
Family	609 (27.87)	888 (40.64)	688 (31.49)
Interpersonal communication	1321 (33.30)	1660 (41.86)	985 (24.84)
Social life & security	286 (18.36)	684 (43.90)	588 (37.74)
Medical care	1327 (31.12)	1266 (29.69)	1671 (39.19)
Occupation & education	1125 (30.17)	1313 (35.21)	1291 (34.62)
Beauty	617 (34.84)	551 (31.11)	603 (34.05)
Self-care	251 (26.96)	390 (41.89)	290 (31.15)

The number of posts with negative emotions in the family, occupation & education, and self-care categories was slightly higher than the number of posts with positive emotions. Therefore, we can infer that users encounter obstacles in family life, employment, and education. The interpersonal communication category had more posts with positive emotions (1321/3966, 33.30%) than with negative emotions (983/3966, 24.84%). Meeting acquaintances is one of the main reasons that people with albinism join OHCs. Finally, there was no significant difference in the proportion of posts with positive (617/1771, 34.84%) or negative (603/1563, 34.05%) emotions in the beauty category, indicating that the user’s mood was relatively stable when talking about makeup or hair coloring, for example.

## Discussion

### Principal Findings

This study explored the topic characteristics and sentiment distribution for an albinism community in the Baidu Tieba OHC from multiple dimensions using LDA, social network analysis, and sentiment polarity analysis. There were 8 hot topics in the communication within the community, of which the daily sharing topic category represented the largest proportion. The social network structure was not stable. The importance of core users was gradually emerging. Emotional differences were demonstrated in distinct topics, implying varying user attitudes and statuses.

### Solve Practical Problems

First, our study demonstrated that users desire to solve practical problems using OHCs. As observed, patients are used to asking for help from people with similar experiences. The increasing proportion of topics on medical care, occupation & education, and beauty was obvious. Among these topic categories, medical care, including prenatal care and diagnosis, was the category that the most users were concerned with, and patients with albinism did not know where to go and what to do, causing anxiety and stress. This suggests that patients would appreciate more professional support, even a cure. In addition, physical defects and social discrimination seriously affected the quality of life of patients with albinism. They continue to demand ways to ease, as much as possible, their daily lives, protecting their rights and interests. Furthermore, users want to relieve social issues by using OHCs to meet people in similar situations. Surprisingly, we found that offline gatherings were mentioned in the original corpora, which is also helpful for further communication between patients. Our results also show that there are relatively close communities of users, which are conducive to the transmission and resolution of information, and the role of core users is gradually increasing across boundaries of smaller communities.

Another survey reported that 62% of respondents recognized the diagnosis, and 69% discussed online information with their physician [61]. Obviously, the use of the internet for health care interactions may represent a necessity for patients with rare diseases to better manage their complex health needs [62]. Furthermore, the creation of online communities for patients and caregivers who share information about their disease may empower them and facilitate participation in clinical trials [63,64]. However, albinism communities do not clearly identify doctors from whom users can seek professional help.

### Improve User Participation and Loyalty

Second, measures should be taken to improve user participation and loyalty in OHCs for albinism. Actual participation in albinism communities is <2% (3977/300,000), which is far less than the number of identified albinism patients. Most users belong to the diving type, indicating that the content in the community does not attract them or they do not have the courage to express opinions in the current environment. Our results show a serious loss of users that has been sustained throughout the past few years. The average number of annual comments continues to decline, and users’ expectations and interest in participating with such communication decrease. It should be noted that this community is likely to disappear in the future, if nothing is done to improve participation. Credibility is a matter of great concern. As commonly agreed, the accuracy and perceived credibility of OHCs is pivotal in facilitating social relationships [65]. A positive correlation also exists between community communication activity and information quality [66]. Therefore, low user participation and loyalty reflect this crisis in the albinism community. The results of the social network analysis show that the influence of core users is gradually expanding, which provides opportunities for professionals to influence the public. However, due to the decline in the overall influence, it is difficult for us to clearly understand the albinism community within this context, especially in the communication environment led by medical staff and specialists.

### Express Feelings

Third, patients with albinism are inclined to express their feelings, especially negative feelings, in OHCs. The combination of topic mining and sentiment polarity analysis revealed the concerns of users and their attitudes towards various issues. The sentiment analysis of the whole corpus showed that 68.42% of posts were emotional; there were 5 topics for which a negative sentiment was more prevalent than a positive sentiment. Therefore, users are used to expressing their feelings through the internet. OHCs provide users with an environment for communication, which is of great importance irrespective of whether the user is a patient or an ordinary user. This is consistent with the research of Delisle et al [67], which summarized 7 different perceived benefits of participating in rare disease support groups, including giving and receiving emotional support and having a place to speak openly about the disease and one’s feelings. Furthermore, membership in online groups can provide those living with long-term conditions with readily available access to self-management and emotional support [68]. The most important positive and negative sentiments were encouragement and worry, indicating that users can get support in OHCs, which will help them overcome difficulties. Negative emotions reflect the worrying situation of patients with albinism and their families. The main issues include a lack of medical-related knowledge, limited amount of national policy on rare diseases, and inferiority caused by the disease. This requires attention from social and medical experts.

### Strengthen the Construction

Finally, the construction of OHCs for albinism should be strengthened to better meet the needs of patients. Based on our analysis of the albinism community, the services from OHCs did not meet the users’ demand. And this contradiction has gradually intensified. Coincidentally, the situation in other albinism communities in China is also serious. Moon Kids Home [69], a relatively professional platform, is currently the largest OHC for albinism in China. Owing to a lack of management, there is a lot of advertising and spam, preventing the platform from functioning normally. The population of patients is small and geographically scattered [70]. It is therefore difficult to organize effective diagnosis and treatment services. We must be aware of the necessity and urgency of building rare disease OHCs. OHCs facilitate patients' access to health care and increase the availability of medical resources. Relevant medical institutions, companies, and government agencies should establish and maintain professional OHCs in the field of rare diseases, which can be single-species or comprehensive, providing a better community environment for patients. OHCs can also effectively assist health care providers in collecting patient information. This information assists providers, informaticians, and online health information entrepreneurs in helping patients and caregivers make informed choices [66]. Users of OHCs acquire knowledge and advice related to health risk evaluation, disease prevention and diagnosis, and treatment suggestions from doctors [65]. In addition, patients may provide self-tracking measurements of vital signs and other biological or behavioral parameters that can be transmitted through the internet and allow for richer information for clinical decisions [71].

In developed countries, organizations focused on rare diseases emerged earlier and developed more rapidly. In the field of albinism, there are already some influential organizations, such as the National Organization for Albinism and Hypopigmentation [72], Albinism Fellowship [73], and Albinism Europe, with patients being able to ask for help through the network. Offline care activities are also carried out, but there is still insufficient space to provide free communication. Given China’s large population, it is generally believed that the country also has the largest population of people affected by rare diseases [74]. Furthermore, government agencies in China have issued the China's First List, which lists 121 rare diseases to facilitate their management [75]. However, the development gap of relevant domestic forums is obvious. Patients with rare diseases and their families are vulnerable in society and deserve more attention and care.

### Implications

The focus of this study is patients with albinism who are easily overlooked and misunderstood by health care providers. OHCs provide the general public with an opportunity to increase their awareness and understanding of the disease. Through topic mining and sentiment analysis, we captured the needs of patients relating to health care, beauty, and making friends. At the same time, we clearly observed obstacles for patients in terms of occupation, education, and social activities, which illustrates the inconvenience caused by physical differences and public discrimination. The role of the albinism community is gradually disintegrating. Obviously, society needs to devote more attention to patients with rare diseases. Relevant health care departments should formulate effective countermeasures based on problems revealed by the results of this study. In addition, this study should also remind us to improve OHCs to satisfy the various needs of patients. We should strengthen psychological counseling via OHCs while improving the living conditions for patients with albinism. Of course, protecting the rights of patients should also be a major priority. All of these require that related agencies, such as medical institutions, companies, and government agencies, establish more professional OHCs for rare diseases based on international experience. In addition, multisector cooperation would allow for the establishment of norms for the creation of OHCs for rare diseases. The research results can only be used as a reference for other rare diseases.

### Limitations

Although findings are based on the conducted analysis, there are still several potential limitations that may encourage further research efforts. First, because there are few OHCs for albinism in China, this study has a limited amount of data, which will have a certain effect on the outcome. Due to the limitations of Baidu Tieba, the fields in which to crawl for data have almost no descriptive indicators for the user. Social network analysis only focuses on the mutual connection of users. Second, although the RAs were trained to mark the corpora to ensure the consistency of the labeling results, the topic labeling process was manual, which might introduce bias to the topic evolution. Third, during the labeling process of supervised learning, part of the corpus had both positive and negative emotion expressions. We mainly used its core sentiment for labeling. This process could cause deviations in sentiment polarity to some extent. However, this situation has little impact on the overall distribution, as the corpora collected were mostly short text. Finally, the sentimental polarity for albinism would change over time due to the change in perception or attitude of the Chinese society towards the patients’ condition. However, such an evolution was not reflected in our study, which could also lead to bias in the analysis and discussion of the sentimental polarity to some extent.

### Conclusions

The combination of topic mining, social network analysis, and sentiment polarity analysis can effectively capture the topics and emotional characteristics of OHC users. This study provides new perspectives for understanding the needs and situations of patients with rare diseases. The albinism community provides a platform for free expression and consultation for Chinese patients with albinism and their families. They have a great demand for medical, inspection, policy, and other related information. Further studies are needed to detect change and the reasons for the sentimental polarity for albinism in OHCs. In addition, research should explore how to strengthen the cooperation of multiple parties to better exert sufficient influence and roles in OHCs. Meanwhile, studies should also be conducted to strengthen the understanding of the social adaptability and psychology of rare disease groups to better learn patient needs.

## Appendix

Multimedia Appendix 1 The total 36 categories obtained from Latent Dirichlet Allocation model, as well as their merging process.

## Abbreviations

CNN: convolutional neural network
LDA: latent dirichlet allocation
LSTM: long short-term memory
NB: naive Bayes
OHC: online health community
RAs: research assistants
SVM: support vector machine
