# Supplementary material for: Determining the Topic Evolution and Sentiment Polarity for Albinism in a Chinese Online Health Community: Machine Learning and Social Network Analysis
Source: JMIR Med Inform. 2020 May 29;8(5):e17813. doi: 10.2196/17813 (PMC7293058; doi:10.2196/17813)
Supplement: Multimedia Appendix 1 [file medinform_v8i5e17813_app1.doc]

**Multimedia Appendix 1:**

**The total 36 categories obtained from Latent Dirichlet Allocation model, as well as their merging process.**

**1. 36 topic distributions generated by LDA model.**

(0, '0.147 * "Newcomer" + 0.040 * "Care" + 0.032 * "Pregnancy" + 0.026 * "Screening" + 0.024 * "Work" + 0.019 * "Diet" + 0.014 * "Friend" + 0.014 * "Hubei" + 0.012 * "Gene" + 0.007 * "Contempt" + 0.007 * "Satisfaction" + 0.007 * " Night" + 0.006 * "Can't stand" + 0.006 * "In" + 0.006 * "Network" ')

(1, '0.053 * "Recommend" + 0.047 * "Rare disease" + 0.034 * "Publish" + 0.019 * "Dyed hair" + 0.015 * "Rare" + 0.015 * "Expert" + 0.015 * "Disease" + 0.009 * "Luoyang" + 0.009 * "True love" + 0.009 * "Porcelain doll" + 0.008 * "Fund" + 0.006 * "Genetics" + 0.006 * "Guarantee" + 0.006 * "Rehabilitation" + 0.006 * "Type" ')

(2, '0.033 * "Makeup" + 0.030 * "Home" + 0.015 * "Little girl" + 0.008 * "Forbid" + 0.008 * "Teach" + 0.007 * "Personality" + 0.007 * "Hello" + 0.007 * "Lost" + 0.007 * "Stable" + 0.007 * "Worship" + 0.006 * "Age" + 0.006 * "Rush" + 0.006 * "Pride" + 0.006 * "House" + 0.006 * "New face" ')

(3, '0.033 * "Love" + 0.015 * "Injury" + 0.013 * "Sunscreen" + 0.011 * " Eye ground" + 0.011 * "Look for a mate " + 0.011 * "Normal " + 0.009 * "Fit" + 0.009 * "Internet" + 0.008 * "Unfortunate" + 0.007 * "Return rate" + 0.007 * "Delicious " + 0.007 * "Read" + 0.007 * "Hairstyle" + 0.006 * "Lighting" + 0.006 * "Good-looking" ')

(4, '0.039 * "Life" + 0.021 * "University" + 0.018 * "Psychology" + 0.012 * "Success" + 0.010 * "Struggle" + 0.010 * "Live" + 0.010 * "Normal person" + 0.007 * " Failure "+ 0.007 *" Creation "+ 0.007 *" Strong "+ 0.007 *" Value "+ 0.006 *" Spirit "+ 0.006 *" Thought "+ 0.006 *" Marriage "+ 0.005 *" Understand "')

(5, '0.075 * "Glasses" + 0.026 * "Beauty" + 0.026 * "Happy" + 0.025 * "Make friends" + 0.020 * "Probability" + 0.020 * "World" + 0.016 * "Identity card" + 0.014 * "Carry" + 0.010 * "Can you" + 0.007 * "Small" + 0.007 * "See clearly" + 0.007 * "Train" + 0.006 * "Stinky" + 0.006 * "Sight glass" + 0.006 * "Concerted effort"')

(6, '0.043 * "Tuina" + 0.040 * "Find" + 0.031 * "Massage" + 0.024 * "Boring" + 0.024 * "Blind" + 0.020 * "Remember" + 0.014 * "Telephone" + 0.008 * "Industry "+ 0.008 *" Chat "+ 0.008 *" Back "+ 0.007 *" Masseur "+ 0.007 *" Program "+ 0.007 *" Seeking "+ 0.007 *" Online shop "+ 0.007 *" Competition "')

(7, '0.020 * "Sunshine" + 0.012 * "New Year" + 0.010 * "Appearance" + 0.009 * "Interview" + 0.009 * "Barrier" + 0.008 * "Stay" + 0.008 * "Appears" + 0.008 * "Girlfriend "+ 0.008 *" Place "+ 0.007 *" Misunderstanding "+ 0.007 *" Full "+ 0.007 *" Gold "+ 0.007 *" End "+ 0.007 *" This time "+ 0.006 *" Eyeball "')

(8, '0.025 * "Gene test" + 0.014 * "Summer" + 0.013 * "Scissor" + 0.012 * "Hobby" + 0.012 * "Hurry" + 0.011 * "Cosmetics" + 0.011 * "Hong Kong" + 0.009 * "Semester" + 0.008 * "Liaoning" + 0.008 * "Disability" + 0.007 * "Sister" + 0.006 * "Military training" + 0.005 * "Workshop" + 0.005 * "Vaccine" + 0.005 * "Small shop" ')

(9, '0.775 * "" + 0.002 * "Kidney" + 0.001 * "Easy to find" + 0.001 * "Phone" + 0.001 * "Bladder" + 0.001 * "Specialty" + 0.001 * "Child" + 0.001 * " Say "+ 0.001 *" Drug "+ 0.001 *" Civil servant "+ 0.001 *" World "+ 0.001 *" Several "+ 0.001 *" Break up "+ 0.001 *" Joke "+ 0.001 *" Side"')

(10, '0.070 * "Marriage" + 0.023 * "Woman" + 0.022 * "Bubbly" + 0.021 * "Black hair" + 0.018 * "This" + 0.015 * "Driving license" + 0.013 * "Exam driving license" + 0.012 * "Wig" + 0.012 * "Congenital" + 0.011 * "Holiday" + 0.008 * "Drive" + 0.008 * "Post" + 0.007 * "Fixed" + 0.006 * "With" + 0.006 * "White" ')

(11, '0.025 * "Symptoms" + 0.019 * "Iris" + 0.018 * "Pigment" + 0.015 * "Congenital" + 0.013 * "Eyeball" + 0.013 * "Strabismus" + 0.013 * "Sunburn" + 0.012 * "Pain" + 0.012 * "Medicine" + 0.010 * "Hair" + 0.010 * "Distress" + 0.009 * "Noun" + 0.009 * "Innate" + 0.008 * "Time" + 0.008 * "Eye" ')

(12, '0.068 * "Recruitment" + 0.045 * "Company" + 0.016 * "Internship" + 0.014 * "Make money" + 0.014 * "Interview" + 0.011 * "Two years" + 0.010 * "Boss" + 0.009 * " Resume "+ 0.008 *" Dating in love "+ 0.008 *" Drinking "+ 0.007 *" Photographing "+ 0.007 *" Sleep "+ 0.007 *" Graduation "+ 0.007 *" Miss sister "+ 0.006 *" Two "')

(13, '0.111 * "Moon child" + 0.059 * "Party" + 0.022 * "Vision aid" + 0.018 * "Answer" + 0.012 * "Age" + 0.012 * "Utility" + 0.010 * "Auxiliary" + 0.010 * "Fee" + 0.009 * "Heilongjiang" + 0.009 * "Helpless" + 0.009 * "Name" + 0.008 * "Before your eyes" + 0.008 * "Distressed" + 0.008 * "Gender" + 0.007 * "Little" " )

(14, '0.022 * "Good-looking" + 0.014 * "Annoying" + 0.012 * "Foreign" + 0.011 * "Tianjin" + 0.010 * "Fair" + 0.010 * "Good luck" + 0.010 * "Life" + 0.009 * "Contact lens" + 0.007 * "See clearly" + 0.007 * "Where to go" + 0.007 * "Touch" + 0.007 * "After" + 0.007 * "Contest" + 0.006 * "Little angel" + 0.006 * "Thick" ' )

(15, '0.120 * "Child" + 0.067 * "Life" + 0.025 * "Parents" + 0.019 * "Mood" + 0.014 * "Face" + 0.014 * "Strong" + 0.014 * "Grow up" + 0.013 * "Home" + 0.013 * "Accept" + 0.013 * "Happiness" + 0.012 * "Inferiority" + 0.012 * "Health" + 0.011 * "Sunlight" + 0.011 * "Fate" + 0.011 * "Coming" ')

(16, '0.196 * "\ u3000" + 0.012 * "story" + 0.004 * "understand" + 0.003 * "Reach" + 0.003 * "with glasses" + 0.003 * "powerful" + 0.003 * "loneliness" + 0.003 * "Love" + 0.003 * "Explanation" + 0.003 * "Talk" + 0.003 * "Name" + 0.003 * "Communication" + 0.003 * "Brother" + 0.003 * "Acacia" + 0.002 * "Network" ')

(17, '0.033 * "Registration" + 0.018 * "Training" + 0.017 * "Traditional Chinese Medicine" + 0.014 * "Volunteer" + 0.009 * "College Students" + 0.009 * "National" + 0.009 * "Contact Information" + 0.009 * "United States" + 0.008 * "Exam" + 0.008 * "Notify" + 0.007 * "Communication" + 0.007 * "Confirmation" + 0.007 * "Number of people" + 0.007 * "Contact" + 0.007 * "Team" ')

(18, '0.059 * "Hair dyed" + 0.045 * "Confused" + 0.043 * "Dyed" + 0.030 * "Son" + 0.018 * "White hair" + 0.015 * "Equity" + 0.014 * "Newcomer " + 0.010 * "United Nations" + 0.010 * "Hair Dye" + 0.010 * "Difference" + 0.008 * "Graduate" + 0.007 * "Graduate school exam" + 0.007 * "Details" + 0.007 * "Swell" + 0.006 * "Blanking" ')

(19, '0.056 * "Lonely" + 0.026 * "Friend" + 0.023 * "Post" + 0.019 * "Long time" + 0.015 * "Acupuncture" + 0.015 * "Habit" + 0.013 * "Tuina" + 0.012 * "Wine "+ 0.011 *" Single "+ 0.011 *" Happy "+ 0.010 *" Mood "+ 0.010 *" Companion "+ 0.009 *" Open "+ 0.008 *" Game "+ 0.008 *" Tonight "')

(20, '0.024 * "Examination" + 0.009 * "Helpless" + 0.007 * "Dew" + 0.007 * "Times" + 0.007 * "Not" + 0.006 * "Blood" + 0.006 * "Key" + 0.006 * "Total Think "+ 0.005 *" Sorry "+ 0.005 *" People "+ 0.005 *" Agree "+ 0.005 *" Regenerate "+ 0.005 *" Arm "+ 0.005 *" Danger "+ 0.005 *" Newborn "')

(21, '0.135 * "Eye" + 0.103 * "Hair" + 0.067 * "Skin" + 0.041 * "Color" + 0.026 * "Brow" + 0.023 * "Birth" + 0.021 * "Daughter" + 0.020 * "Worry "+ 0.018 *" Black "+ 0.013 *" Sun "+ 0.013 *" Childhood "+ 0.012 *" Cute "+ 0.012 *" Lashes "+ 0.012 *" Son "+ 0.008 *" Ray "')

(22, '0.250 * "Friend" + 0.201 * "Job" + 0.047 * "Job search" + 0.021 * "Thanks" + 0.020 * "True" + 0.018 * "Experience" + 0.016 * "Back" + 0.014 * " Graduation "+ 0.012 *" Mutual "+ 0.011 *" Suggestion "+ 0.006 *" Response "+ 0.006 *" Business "+ 0.005 *" Interview "+ 0.004 *" Desire "+ 0.004 *" Self introduction "')

(23, '0.084 * "Happiness" + 0.043 * "Girlfriend" + 0.037 * "Happy New Year" + 0.028 * "Dating" + 0.019 * "Man" + 0.013 * "Disability Certificate" + 0.009 * "Misery" + 0.008 * "Report" + 0.008 * "Life" + 0.008 * "Thinking of success" + 0.007 * "Cure" + 0.007 * "Shang" + 0.005 * "Health" + 0.005 * "Miss" + 0.005 * "Own" )

(24, '0.019 * "Hello " + 0.015 * "Aggrieved" + 0.014 * "Insomnia" + 0.010 * "Reading" + 0.009 * "Estimated" + 0.009 * "Handsome " + 0.009 * "Companion" + 0.009 * " Foreigner "+ 0.009 *" Work "+ 0.008 *" Competent "+ 0.008 *" Professional "+ 0.008 *" Applicant "+ 0.007 *" Wrong "+ 0.007 *" Green hand "+ 0.007 *" Ha-ha "')

(25, '0.012 * "Baby" + 0.012 * "Passing by" + 0.010 * "Sunshine" + 0.009 * "Kind friends" + 0.008 * "Landscape" + 0.008 * "Impulse" + 0.007 * "World" + 0.007 * " Angle "+ 0.006 *" Stranger "+ 0.006 *" Butterfly "+ 0.006 *" Chinese "+ 0.006 *" Fuzzy "+ 0.006 *" Smile "+ 0.006 *" True love "+ 0.005 *" Social "')

(26, '0.078 * "Hospital" + 0.066 * "Examination" + 0.049 * "Treatment" + 0.045 * "Doctor" + 0.024 * "Gene" + 0.014 * "Girlfriend" + 0.013 * "Confirmed" + 0.012 * "Check "+ 0.011 *" Newborn "+ 0.010 *" Amniotic fluid "+ 0.009 *" Technology "+ 0.009 *" Soon "+ 0.008 *" Partner "+ 0.008 *" Gene check "+ 0.008 *" Piercing "')

(27, '0.012 * "Idea" + 0.011 * "Invisible" + 0.011 * "Emotion" + 0.010 * "Despair" + 0.009 * "Domestic" + 0.009 * "Magnifier" + 0.008 * "Wings" + 0.008 * "Oscillate"+ 0.007 *" Thanks very much "+ 0.007 *" Remember "+ 0.006 *" Get along "+ 0.006 *" After marriage "+ 0.006 *" Optometry "+ 0.005 *" Tutor "+ 0.005 *" Expectation "')

(28, '0.091 * "Happy" + 0.013 * "Singing" + 0.013 * "Growth" + 0.011 * "True" + 0.011 * "Good" + 0.010 * "Future" + 0.010 * "Ideal" + 0.009 * "Peace "+ 0.009 *" Love "+ 0.008 *" Happy "+ 0.007 *" Home "+ 0.007 *" Valentine "+ 0.006 *" Record "+ 0.006 *" High school "+ 0.006 *" Good night "')

(29, '0.071 * "Exchange" + 0.045 * "Follow" + 0.039 * "Home" + 0.036 * "WeChat" + 0.034 * "Public welfare" + 0.032 * "Recruitment" + 0.016 * "Warm" + 0.015 * "Friends "+ 0.012 *"Long time no see "+ 0.011 *" Discussion "+ 0.009 *" Depressed "+ 0.006 *" Living "+ 0.006 *" Bland "+ 0.005 *" Cozy "+ 0.005 *" Stable "')

(30, '0.146 * "Vision" + 0.040 * "Patient" + 0.023 * "Genetic" + 0.021 * "Eye" + 0.021 * "Skin" + 0.017 * "Gene" + 0.014 * "Myopia" + 0.012 * "Eyeball "Tremor" + 0.011 * "chromosome" + 0.011 * "retina" + 0.010 * "Abnormal" + 0.009 * "caused" + 0.009 * "Melanin" + 0.009 * "Glasses" + 0.009 * "Eye" ')

(31, '0.015 * "Teacher" + 0.015 * "School" + 0.014 * "Classmate" + 0.013 * "Learning" + 0.012 * "Fear" + 0.011 * "Discrimination" + 0.010 * "Give up" + 0.010 * "University "+ 0.009 *" Future "+ 0.009 *" College entrance examination "+ 0.009 *" Parents "+ 0.008 *" Primary school "+ 0.008 *" Worry "+ 0.008 *" Vision "+ 0.008 *" Knowledge "')

(32, '0.068 * "Moon Kids Home" + 0.037 * "Consultation" + 0.032 * "Employment" + 0.031 * "Patient" + 0.026 * "Family" + 0.017 * "National" + 0.017 * "Business" + 0.017 * "Related" + 0.016 * "Institution" + 0.014 * "Disability" + 0.013 * "Concern" + 0.013 * "Graduate" + 0.012 * "University" + 0.011 * "Prenatal diagnosis" + 0.010 * "Center" ')

(33, '0.113 * "Patient" + 0.027 * "Child" + 0.020 * "Surgery" + 0.020 * "Family" + 0.019 * "Information" + 0.018 * "Parent" + 0.018 * "Assistance" + 0.016 * " Found "+ 0.016 *" Moon Kid's Home "+ 0.015 *" Object "+ 0.014 *" Website "+ 0.013 *" Proof "+ 0.012 *" Medical "+ 0.011 *" Vision Assistance "+ 0.011 *" Social "' )

(34, '0.201 * "\ n" + 0.014 * "skin" + 0.010 * "normal person" + 0.010 * "patient" + 0.008 * "melanin" + 0.008 * "disease" + 0.007 * "lack" + 0.007 * "Name" + 0.006 * "Gene diagnosis" + 0.006 * "Lens" + 0.006 * "Look in the mirror" + 0.005 * "Prenatal" + 0.005 * "Science" + 0.005 * "Sunlight" + 0.005 * "Feature" ')

(35, '0.037 * "Social" + 0.032 * "Group" + 0.028 * "Disabled" + 0.015 * "Development" + 0.009 * "Environment" + 0.008 * "Disability" + 0.008 * "Integration" + 0.007 * " Platform "+ 0.006 *" Origin "+ 0.006 *" Education "+ 0.006 *" Suggestion "+ 0.005 *" Related "+ 0.005 *" Function "+ 0.005 *" Psychology "+ 0.005 *" Bus "')

**2. Topic labeling results for 36 LDA categories.**

| Category number | Topic label |
| --- | --- |
| 0 | Communication, Genetic testing |
| 1 | Hairdressing, Genetics, Recovery |
| 2 | Make-up, Life |
| 3 | Make-up, Affection, Protection |
| 4 | Livelihood, Emotion |
| 5 | Friend |
| 6 | Occupation |
| 7 | Life, Employment |
| 8 | Cosmetic, Genetic testing |
| 9 | Medicine |
| 10 | Driving license, Beauty |
| 11 | Medicine, Protection |
| 12 | Occupation |
| 13 | Society, Instrument |
| 14 | Life, Society |
| 15 | Family |
| 16 | Life, Friend, Affection |
| 17 | Occupation, Education |
| 18 | Hairdressing, Graduate school exam, Social security |
| 19 | Life, Friend, Occupation |
| 20 | Life, Examination |
| 21 | Organ, Family |
| 22 | Friend, Employment |
| 23 | Affection, Society, Self-care |
| 24 | Friend, Employment |
| 25 | Communication, Society |
| 26 | Genetic testing, Treatment |
| 27 | Life, Self-care |
| 28 | Life |
| 29 | Communication |
| 30 | Genetic testing, Heredity, Vision |
| 31 | School, Society |
| 32 | Community, Family, Society |
| 33 | Medicine, Community, Society |
| 34 | Medicine |
| 35 | Society |

**3. Merging concepts and semantics to generate final topic classification results.**

| Number | Topic name | Topic label |
| --- | --- | --- |
| 1 | Daily sharing | Life, Affection, Livelihood, Emotion |
| 2 | Family | Family |
| 3 | Interpersonal communication | Communication, Friend, Community |
| 4 | Social life & security | Society, Driving license, Social security |
| 5 | Medical care | Genetic testing, Genetics, Recovery, Protection, Medicine, Examination, Organ, Treatment, Heredity, Vision, |
| 6 | Occupation & Education | Occupation, Employment, Education, Graduate school exam, School, |
| 7 | Beauty | Hairdressing, Make-up, Cosmetic, Beauty, |
| 8 | Self-care | Self-care, Instrument |
